# Little All Children in Focus (Little ACF), evaluation of a parental support program for parents of children aged 1–2 years: study protocol for a randomized controlled trial

**DOI:** 10.1186/s13063-023-07212-4

**Published:** 2023-03-13

**Authors:** Lisa Blom, Anna Edenius, Pia Enebrink, Anders Hjern, Sven Arne Silfverdal, Johan Åhlén, Malin Bergström, Lene Lindberg

**Affiliations:** 1 Department of Global Public Health, Karolinska Institutet, Stockholm, Sweden; 2 Department of Medicine, Karolinska Institutet, Stockholm, Sweden; 3 Department of Clinical Neuroscience, Karolinska Institutet, Stockholm, Sweden; 4 Department of Clinical Sciences, Umeå University, Umeå, Sweden

**Keywords:** Parent program, Parent group, Parent support, Universal, Positive psychology, Health promotion, Parental self-efficacy, Emotional regulation, Child health care, Infants

## Abstract

**Background:**

Child health and development can be promoted by strengthening and supporting parents. Research on parental support programs based on positive psychology and a health-promoting approach aimed at all parents, and in particular parents of infants is limited. All Children in Focus (ACF) is a parental support program that has been evaluated in a randomized trial in parents of children 3–12 years. The ACF is based on health promotion aiming to increase parents’ confidence and child’s well-being. In the current study, we will study the effects of a revised version of the ACF called *Little ACF* adapted to parents with children aged 1–2 years.

**Methods:**

The study includes a randomized controlled trial (RCT) taking place at several Child Health Centers (CHCs) in Sweden. The RCT will evaluate the efficacy of *Little ACF* (intervention) in comparison with four digital lectures about child development and parenting (active control). Parents are recruited at the 10-, 12-, or 18-month visits to CHC by CHC-nurses. Data to assess changes in parental competencies and child socio-emotional development are collected through online questionnaires completed by parents at five time points: baseline, post-intervention, after 6 and 12 months, and when the child is 3 years old.

**Discussion:**

The paper describes a study protocol of a randomized controlled trial evaluating the effects of a parental support program during infancy. Several issues related to the methodology and implementation are discussed.

**Trial registration:**

ClinicalTrials.gov NCT05445141. Registered on 6 July 2022.

## Administrative information

**Table:** 

**Title {1}:**	Little All Children in Focus (Little ACF), evaluation of a parental support program for parents of children aged 1–2 years: study protocol for a randomized controlled trial
**Trial registration {2a and 2b}:**	Registered at ClinicalTrials.gov on 6 July 2022, Study identifier: NCT05445141
**Protocol version {3}:**	Version 2 of 21 February 2023
**Funding {4}:**	This research is funded by The Swedish Research Council 2021–06447 and by grants provided by Region Stockholm RS2020-0732
**Author details {5a}:**	Lisa Blom: Karolinska Institutet, Sweden. Anna Edenius: Karolinska Institutet, Sweden. Pia Enebrink: Karolinska Institutet, Sweden. Anders Hjern: Karolinska Institutet, Sweden. Sven Arne Silfverdal: Umeå University, Sweden. Johan Åhlén: Karolinska Institutet, Sweden. Malin Bergström: Karolinska Institutet, Sweden. Lene Lindberg: Karolinska Institutet, Sweden
**Name and contact information for the trial sponsor {5b}:**	Investigator initiated trial; Center for Epidemiology and Community Medicine, Karolinska Institutet & Child Health Services, Region Stockholm. Lene Lindberg (Principal Investigator) lene.lindberg@ki.se
**Role of sponsor {5c}:**	Investigator initiated trial. The funders had no role in design of the study, data collection, analysis, interpretation of data or in writing the manuscript

## Introduction

### Background and rationale {6a}

It is of utmost importance and interest for society to promote children’s development and well-being by creating good opportunities for parents. One way to strengthen parents is through programs that promote children’s health or prevent ill health [1]. Most prevention programs offered to parents are primarily aimed at parents with acting-out children [2]. Universal programs delivered to all parents based on positive psychology [3] and health promotion [4] have not been studied to the same extent [5, 6]. Positive psychology was introduced by Seligman in the 1990s and focuses on various components that relate to well-being; i.e., positive emotions, engagement, relationships, meaning, and achieving one’s goals [7, 8]. Within positive psychology, the concept of “self-efficacy” — belief in one’s own ability — has also been described as an important part of well-being [9].

Health promotion is described by the World Health Organization (WHO) as a process where the individual can take control and improve their health through participation and commitment [10]. Well-being is also an important concept within health promotion although ill-defined. According to positive developmental psychology, well-being during infancy is promoted through the development of emotional regulation [11]. Emotional regulation is developed in interaction with parents through mirroring, putting words to the child’s feelings, and by showing positive feelings for the child. Parents need to support each other and be role models for the child [12, 13]. This is also in line with what is presented in the theory of coparenting, which describes how parents need to develop mutual trust and agree on the distribution of responsibilities, values about child rearing, and how conflicts can be handled [14]. A positive parental relationship is an important component for the child’s well-being according to positive developmental psychology [12].

In a meta-analysis of studies that evaluated support (mostly prevention) to parents with children between 0 and 7 years of age, it emerged that parental support including communication of feelings showed effects on parents’ behavior. Opportunities to practice skills showed greater effects on parents’ behavior and child externalizing problems [2]. The largest effects for parents were increased parental efficacy and for children a reduction in internalizing problems [2].

The state of evidence for universal parenting programs [1] is still limited and programs aimed at parents with infants are unusual, despite the fact that parenthood in this period may contain many challenges [15]. In summary, there is a great need to develop and evaluate promotional efforts aimed at parents with infants.

All Children in Focus (ACF) is a parental support program based in health promotion that was developed and evaluated in Sweden for parents of children aged 3–12 years [16]. ACF is developed based on research and was developed in dialog with parents and professionals. A randomized controlled trial (RCT) found that ACF 3–12 years increased parents’ confidence and well-being of the child [16]. Here, we are testing a revised version of ACF called *Little ACF* in Swedish that is adapted to parents with children aged 1–2 years.

### Objectives {7}

The aim of the study is to increase the knowledge on the effects of *Little ACF* on parents and children.

Our specific research questions are how *Little ACF* can:

- Strengthen strategies of emotional regulation in parents,
- Promote parenting practices,
- Promote child socio-emotional well-being,
- Lead to the reduction of child behavior problems (externalizing/internalizing), and
- Engage and retain parents in groups?

### Trial design {8}

The study includes a two-arm parallel superiority RCT to evaluate the efficacy of *Little ACF* in comparison with four digital lectures covering child development and parenthood. The allocation ratio applied was 1:1.

## Methods: participants, interventions, and outcomes

### Study setting {9}

The study is conducted in collaboration with thirteen Child Health Centers (CHCs) in Sweden. The included CHCs had registered interest to the request that was sent out to all CHCs in the Stockholm region for participation in the study Little ACF. To be included, the management at each CHC also had to appraise that the CHC had sufficient capacity in terms of employed nurses to ensure participation. The included CHCs are situated in the region of Stockholm, and a few of them are located in commuting municipalities of Stockholm and others in small towns, altogether representing both urban and rural areas. Additional CHCs might be included in the study before recruitment is finished. The specific names of the included CHCs are not presented to reduce the risk of bias in the assessment of the study.

Today, all families in Sweden are offered ten individual appointments at the local CHC before the child turns 18 months [17]. Parents are often invited to participate in some form of parenting groups when the child is between 2 and 11 months, but the content of these groups varies greatly, and the efficacy of those groups has never been evaluated scientifically. The content is usually focused more on the child’s development than on parenting [18] and the groups mainly reach mothers [19].

Given that almost all families with young children attend the Swedish CHCs, it is a highly relevant arena for the development and evaluation of additional structured parenting support efforts [17] and a natural place for both recruitment and implementation.

### Eligibility criteria {10}

#### Inclusion criteria

- Parents of children in the age group 1–2 years who are registered at any of the included CHCs

#### Exclusion criteria

- Parents in need of interpreters

### Who will take informed consent? {26a}

For the group leaders, a preliminary consent via phone or email was provided in relation to registering to the training. Written consent was then collected at the first training occasion.

At the child’s ordinary 10-, 12-, or 18-month visit at the CHC, the CHC-nurse informs the parents about the study orally and provides them with an information flyer about the study. Parents who declare interest in participation receive an email with a link to a study-specific website. On the website, they sign the consent digitally if they wish to participate and then complete a digital baseline questionnaire. All caregivers to the child are encouraged to participate.

## Interventions

### Explanation for the choice of comparators {6b}

Parents who are assigned to the control group are offered four recorded digital lectures that are available for about four weeks. The lectures are 10 to 19 min long and include age-appropriate information about parenthood and child development. The lectures are information-based without providing parenting strategies. The themes are (1) food and eating, (2) sleep, (3) child accidents and infections, and (4) curiosity as a driving force in child development. A waitlist control design was considered as an alternative to the four recorded digital lectures, but this was not regarded as possible due to the long follow-up time of the participants and the narrow timespan of when the intervention is meant to take place (when the children are 12–24 months). The decision to offer lectures to the control group was made based on suggestions from the group leaders from the pilot study to increase the chances of recruiting and retaining participants in the control group by offering some kind of additional activity on top of usual care since no reimbursement is provided to the participants and since wait-list control group was not possible.

### Intervention description {11a}

#### The intervention *Little ACF*

The program *Little ACF* is based on the parental support program ACF for parents to 3–12-year-olds [20]. *Little ACF* was developed focusing on children 1 to 2 years, a period in life when families go through many changes [15]. During this year, the child usually starts preschool, parents go back to work or studies, and the support from CHC gradually declines with the visits becoming less and less frequent. During this period, fathers are often on parental leave and may therefore be more likely to participate in parental groups.

**Fig. 1:**
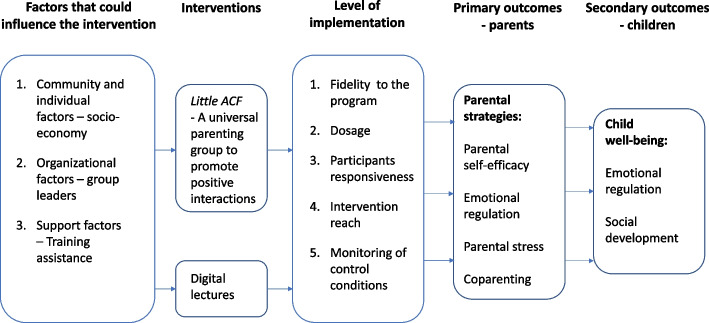
Logic model for how *Little ACF* can lead to outcomes in parents and children

The adjustment of the program ACF to *Little ACF* has been realized through a collaboration between CHCs in Region Stockholm, the unit PLUS in Stockholm municipality, and the Center for Epidemiology and Community Medicine (CES) in Region Stockholm. The final version of *Little ACF* was developed and finalized by CES. The content of *Little ACF* is based on research on parenthood and child development, the United Nations Convention on the Rights of the Child, and expressed needs and wishes of parents of small children. The material was tested in a pilot study in 2021 when 10 group leaders were trained and led five parent support groups. The pilot led to a revision of the material including a stronger focus on emotional regulation and secure relationships. Figure 1 shows a logic model of how *Little ACF* is assumed to lead to outcomes in parents and children.

The program *Little AFC* consists of four group meetings with four different themes (described in Table 1). The theoretical foundation is positive psychology including research about attachment, parenting and coparenting, emotional regulation, and observational learning [15]. Each group is led by two group leaders of which one is a CHC-nurse, and the other is a group leader from the municipal or civic society that works with parents of small children. The group meetings are scheduled to 60 min and the parents can thereafter stay and discuss and socialize for 30 min. The parents are allowed to bring their children to the group meetings due to the young age of the children.

**Table 1:** Summary of the content of the group meetings in *Little ACF*

**Meeting 1** **Children are different**	The content of the first meeting deals with children’s innate temperament, how it can affect parenting, and how parents can strengthen self-esteem and security in their children. The meeting starts with a welcome session where participants present themselves and are provided with some practical information. The group leaders introduce *Little ACF*, explain the goal with the meetings and present the content of the current meeting. The themes for the first meeting are (1) temperament, (2) sensitive parenting, and (3) putting words to the child’s feelings. Theoretical elements are alternated with specifically designed illustrations, role plays, and group discussions. The meeting ends with an introduction to the example to try out at home that focuses on putting words to the child’s feelings. The printed material is distributed to the parents
**Meeting 2** **Being together**	The content of the second meeting focuses on infant development and how parents can regulate infant’s emotional expressions. The second meeting starts with the group leaders introducing the content of the meeting and a discussion about examples tested at the home. The themes of the second meeting are (1) autonomy, curiosity, and emotional expression; (2) challenging situations, (3) make everyday work; and (4) validate, explain, repeat, and distract. Theoretical elements are alternated with illustrations and group discussions. The meeting ends with an introduction to the example to try out at home that focuses on validation, explain, repeat, and distraction. The printed material is then distributed
**Meeting 3** **Regulation of parental emotions**	The content of the third meeting focuses on challenging parental feelings and how parents can regulate their own emotions and maintain sensitive parenting. The third meeting starts with an introduction to the topics and a follow-up on examples tested at home. The themes of the third meeting are (1) emotions are contagious, (2) stress, (3) the emotional curve, and (4) stop-think-act. Theoretical elements are alternated with illustrations and group discussion. The meeting ends with the introduction to the final home exercise focusing on testing the strategy Stop-think-act. The printed material is thereafter distributed
**Meeting 4** **Secure relations**	The last meeting focuses on the importance of secure relations for both parents and children, about separations, and how parents can help the child feel safe with other significant adults. The fourth meeting starts with the day’s topics and a follow-up on what has been tested at home. The themes of the fourth meeting are thereafter (1) a network of secure relations, (2) being away from each other, (3) catching the golden moments, and (4) closure. Theoretical elements are alternated with illustrations and group discussions. The meeting ends with group leaders informing about other available support to parents in their local community. Parents are provided the printed material including a list of available parental support

The content is based on role plays and discussion topics. The manual for the group leaders is structured similarly for all four meetings with clear instructions on what is expected by the group leaders at each stage for example when questions should be posed. The three first meetings end with an example to try at home and the following meeting starts with discussing these examples. The examples are designed to stimulate reflection among the parents and to encourage them to test new positive approaches in the interaction with their child. Parents will be distributed a printed material at each meeting that will also be available on a web-portal. The parents can use the web-portal between the meetings to read and share the content to the other parent or other significant adults around the child.

### Criteria for discontinuing or modifying allocated interventions {11b}

Participants are allowed to leave the study at any time without having to disclose the reason for doing so. If a participant decides to leave the study, data that have been collected until that point will be included in the analysis.

### Strategies to improve adherence to interventions {11c}

No reminders are sent out for the group meetings, or the lectures and no reimbursements are provided to the participants in the study. Group leaders fill out a questionnaire after each session about parental engagement and the group leaders adherence to the manual. Supervision to the group leaders is offered regularly during the trial to discuss enabling and aggravating circumstances during the sessions.

### Relevant concomitant care permitted or prohibited during the trial {11d}

Participants in both groups are attending care as usual with the ordinary visits to CHC. There are no restrictions on care or attendance to other parental interventions during the trial.

### Provisions for post-trial care {30}

No post-trial care is provided.

### Outcomes {12}

Data are collected at five time points (Table 2): baseline (T1 – infant age 12–18 months), post-intervention (T2 – infant age about 14–20 months), after 6 months (T3 infant age 18–24 months), after 12 months (T4 – infant age 24–30 months), and in conjunction with the CHC visit when the child is 3 years old (T5). The data are collected through questionnaires to the parents. The baseline questionnaire contains background questions on the age and sex of the child and the parent and questions on parental educational level, country of birth, and family composition as well as several validated instruments that are repeated in the follow-up questionnaires.

**Table 2:** Time points for the specific outcome measures and intervention assessments

	**Time points**					
**Assessments**	**T1**	**Intervention**	**T2**	**T3**	**T4**	**T5**
***Parents***						
Baseline variables	X					
**Parental strategies:**						
Parental self-efficacy	X		X	X	X	X
Parent emotional regulation	X		X	X	X	X
Coping with child negative emotions	X		X	X	X	X
Parenting stress	X		X	X	X	X
Coparenting relationship	X		X	X	X	X
***Child well-being (through assessments of parents)***						
Child’s social and emotional development	X		X	X	X	X
Psychological and behavioral problems						X
***Group leaders***						
Checklists group leaders		X X X X				

#### Primary outcome

The primary outcome is five aspects of parenting strategies, measured at all five time points of the study (T1, T2, T3, T4, and T5). Parenting self-efficacy (PSE) [16, 20] is measured with 48 items that are assessed with an 11-point Likert scale from 0 (completely disagree) to 10 (fully agree) using a modified version of *Tool to Measure Parenting Self-Efficacy* (TOPSE) [21].

The emotional regulation of the parent in relation to the child is measured using the *Parent Emotion Regulation Scale* (PERS) developed by Pereira et al. [22]. The PERS consists of 20 questions that are to be answered on a scale from 1 (never or almost never) to 5 (always or almost always). For the purpose of our study, questions nr 1, 11, and 18 were removed based on a validation study of the PERS that took place prior to the study (Manuscript in preparation).

How parents handle children’s negative emotions is assessed using the *Coping with Toddlers’ Negative Emotion Scale* (CTNES) [23]. In its original version, 12 situations are described to which the parents should assess the likelihood of using 6–7 different strategies to solve each situation. The likelihood assessment ranges from 1 (not likely at all) to 7 (very likely). For this study, 8 situations (items 3–10) were selected based on the analysis from the validation of the Swedish version (Manuscript in preparation).

Parental stress is assessed using the scales *incompetency* and *role limitation* from the *Swedish Parenting Stress Questionnaire* (SPSQ) [24]. The items are assessed with 5 response alternatives.

Five questions inspired by the *Coparenting Relationship Scale* [25] are used to assess the quality of parents’ coparenting relationship. The answers range from 1 (not true at all) to 5 (very true) on a 5-point scale.

#### Secondary outcome for children (based on parents’ assessments)

Effect from the program on the well-being in terms of social and emotional development of the child is expected at T3, T4, and T5.

The child’s social and emotional development is measured at T1, T2, T3, T4, and T5 with the *Ages & Stages Questionnaires – Socio-Emotional Second Edition* (ASQ:SE-2) [26]. It consists of 29–35 questions depending on the age, with 3-point scale response alternatives ranging from “often or always,” “sometimes,” to “seldom or never”. The parent can also indicate whether they worry about the behaviors. The ASQ:SE-2 ends with two open-ended questions.

To assess psychological and behavioral problems in the child, we use parents’ assessments of the *Strengths and Difficulties Questionnaire* (SDQ) [27] at the child age 3 years. The SDQ relates to the child’s behavior over the last 6 months and includes 25 questions that are answered on a 3-point scale with the alternatives “not true at all,” somewhat true,” and “completely true.” The SDQ is only measured at T5.

#### Implementation and other factors that could affect the outcomes

Parents’ attendance at the group meetings is registered by the group leaders. The follow-up questionnaires also contain questions to the parents in the intervention group about how they have used the content from the group meetings, whether they have tested the content at home, and if they found it useful. The parents in the control group are asked if they viewed the digital lectures and if they considered them meaningful.

The group leaders complete a checklist after each meeting including questions about if they had time for the different modules, how the home exercises worked, how committed the parents were during the meeting, and how much use they had of their group leading skills. The group leaders are also asked whether they perceived that they had sufficient knowledge about the content, any challenges that came up and their perspective on the material for the meetings in addition to how many parents and children were present.

### Participant timeline {13}

**Fig. 2:**
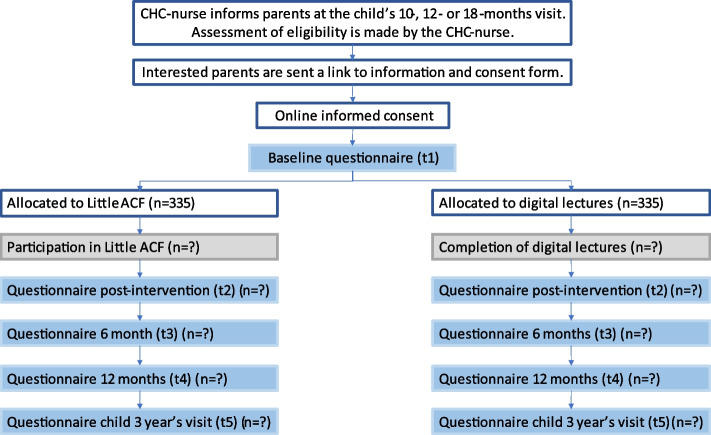
Flowchart of the study and the participant timeline

Figure 2 gives an overview to the participant timeline in the study.

### Sample size {14}

#### Power calculation

A previous RCT of the program ACF for parents of children 3–12 years of age showed an effect size (Cohen’s *d*) of 0.30 for parental self-efficacy [16]. We expect a slightly smaller effect (*d* = 0.25) because this study compares *Little ACF* with lectures while the previous study had a waitlist control group. A total of 251 participants in each group are therefore needed with a significance level of 95% (*p*-value 0.05) and a power of 80%. A drop-out of 25% is expected resulting in the need to include 670 families in total. Each group consists of parents to 5–8 children so about 60–70 groups are needed. Each group leader is expected to lead at least three groups which means that about 25 group leader pairs are needed in total.

### Recruitment {15}

#### Group leaders

The recruitment of group leaders started with an information letter to all managers at potential health care facilities. Most recruitment took place at the CHCs. If a CHC-nurse was interested to participate, the nurse was required to contact a potential group leader partner from the municipality or another facility from the civic society, e.g., the Swedish church. The group leaders had to register for the training in pairs (this was a prerequisite from the project team). Potentially interested group leaders were invited to a digital workshop. The next step was a digital workshop to all those who had registered to the training where the information became more detailed. The workshop started with a presentation followed by the opportunity for the coming group leaders to ask questions.

The group leaders participated in three days of training sessions scheduled for every other week to allow for them to run groups in between. The training included theory and practical exercises and supervision is offered monthly during the trial. The group leaders are expected to lead in total at least three groups during the trial.

#### Parents

Parents are asked for participation by the CHC-nurse at the child’s ordinary 10-, 12-, or 18-month visit at CHC. The recruitment of parents started on August 1^st^, 2022, when the study had been approved by the Swedish Ethical Review Authority (Dnr 2022–02,603-01).

## Assignment of interventions: allocations

### Sequence generation {16a}

Families are allocated by simple random sampling into either arm of the study using the platform CSAM MedSciNet Clinical Trial Framework (CSAM). When the CHC-nurse has recruited 10–16 families, 50% of the families will be assigned to the intervention group and 50% to the control group.

### Concealment mechanism {16b}

Use of a validated password website CSAM will ensure concealment.

### Implementation {16c}

When twice the number of families needed for a full parental group have completed baseline questionnaires, the CHC-nurse uses the platform CSAM to randomize the families into the two arms of the study. CSAM performs the randomization without any possibility for the CHC-nurse to intervene. Information about allocation is sent automatically to the families by CSAM after randomization. Links to the digital lectures are included in the email to the control group. Thereafter, the parents in the intervention group receive information about the time and place for the group meetings from the CHC-nurse.

## Assignment of interventions: blinding

### Who will be blinded {17a}

Neither participants nor group leaders are blinded because of the nature of the intervention and for ethical reasons. Everyone is aware of the two groups (Little ACF and digital lectures). Researchers are blinded to intervention allocation (Little ACF or digital session) during statistical analyses of the primary outcome.

### Procedure for unblinding if needed {17b}

Unblinding for researchers will be done in the study after statistical analyses of the primary outcome.

## Data collection and management

### Plans for assessment and collection of outcomes {18a}

The platform CSAM is used for the collection of data and for study management overall. The platform is familiar to the CHC-nurses and is used in their regular work. All outcome assessments on the parents and the children are made by questionnaires answered by parents. Participating parents are sent links to the questionnaires via the CSAM platform and can answer to the questionnaires electronically on their own smartphone, computer, or tablet. The information is stored on the platform CSAM, downloaded to Excel by the research team, and stored on secure servers at Karolinska Institutet.

The group leader checklists are filled in by hand on paper after each group meeting, and thereafter either scanned and emailed or sent by post to the study coordinator.

### Plans to promote participant retention and complete follow-up {18b}

If a participant has not completed a questionnaire, up to three reminders are sent out if needed. The first one after 3 days, the second one after 10 days, and the last one after 17 days. The group leaders from the municipality or civil society will telephone non-responders after the third automatic reminder.

### Data management {19}

All data are handled in line with the General Data Protection Regulation (GDPR) and procedures of safe handling and storage. The output of the questionnaires including the electronic informed consent of the parents will be stored in the platform CSAM and downloaded to password-protected computers. Access to the data is strictly restricted to the research team. The signed informed consents of the group leaders will be stored in a locked cabinet at the department of global public health at Karolinska Institutet. All research data will be archived for 10 years and thereafter destroyed.

### Confidentiality {27}

The names of the parents and personal identification are replaced by a study ID in the data set. Only the research group has access to the study IDs.

### Plans for collection, laboratory evaluation, and storage of biological specimens for genetic or molecular analysis in this trial/future use {33}

No biological specimens are collected in this study.

## Statistical methods

### Statistical methods for primary and secondary outcomes {20a}

The SPSS and R software will be used for all statistical analyses. Data will be tested for assumptions of parametric statistics. Examinations of pattern and amount of missing data will be performed and if appropriate treated by using relevant estimates. Statistical tests will be two-sided, and the level of significance will be set to 0.05.

Family and infant characteristics will at baseline be summarized overall and by trial arms, with means and standard deviations or medians and ranges for continuous variables. Numbers and percentages will be used for categorical variables. Summarized baseline measures regarding parental strategies and child well-being will also be shown. Any differences between groups at baseline will be examined with *t*-test for independent samples, chi-square test, or Mann–Whitney *U* test.

Fidelity measured as the group leader’s adherence to the program and parents’ participation in *Little ACF* or digital sessions will be summarized with descriptive statistics. Researchers performing statistical analyses of the primary outcome will be blinded to intervention allocation (*Little ACF* or digital session). Measures of effect size will be estimated to assess intervention effects. Hierarchical regression model will be used for assessing primary and secondary outcomes. Linear mixed regression model will be used for continuous data and generalized mixed models for binary and ordinal data. Intervention effects will be assessed for the primary outcome at the post-intervention and at the follow-ups with control for baseline scores. For the secondary outcome, intervention effects will be measured at the follow-ups with control for baseline and post-intervention scores.

### Interim analyses {21b}

No interim analyses are planned.

### Methods for additional analyses (e.g., subgroup analyses) {20b}

Demographic information about the participants will be presented descriptively to describe the outreach and uptake of the intervention. Information about the implementation of the program and the participation of the families will be used to assess the exposure of the program.

### Methods in analysis to handle protocol non-adherence and any statistical methods to handle missing data {20c}

The results will be analyzed with an intention-to-treat approach and per protocol. Missing data will be analyzed and decisions on imputations for the missing data in the study will be discussed within the research team based on that information.

### Plans to give access to the full protocol, participant level-data and statistical code {31c}

The datasets analyzed during the current study and statistical code are available from the corresponding author on reasonable request and with ethical permission under Swedish law for secondary data analysis, as is the full protocol.

## Oversight and monitoring

### Composition of the coordinating center and trial steering committee {5d}

The study is designed, performed, and coordinated by a research team at the Karolinska Institutet and Region of Stockholm. The principal investigator and one senior researcher from the research group are responsible for the overall supervision of the trial. The study coordinator who is conducting her PhD in the project is responsible for the coordination of the research duties.

Three working groups including stakeholders from the CHCs and other organizations have been created. One working group is responsible for the development of the material including pilot testing the material and developing the manual and the layout. Another group is responsible for the it-platform including the questionnaire and measurement instruments. The third one is responsible for the training of group leaders and for providing monthly supervision and mentoring throughout the trial. The three working groups meet every other week to discuss and allocate tasks and solve any issues that come up.

The trial steering group includes all the researchers, key persons from Region Stockholm, and Stockholm County board. The steering group has meetings when needed.

### Composition of the data monitoring committee, its role and reporting structure {21a}

No data monitoring committee has been appointed to this study since the intervention is considered to have a low risk of negative consequences and is of a non-invasive character. The intervention is directed to the parents and the outcomes in the children are only captured through parental reports.

### Adverse event reporting and harms {22}

Previous research on the program ACF for parents of children 3–12 years old has not resulted in any adverse events. Any potential issues that come up during the group meetings will in the first place be handled by the group leaders, all of them have training and experience of running parental groups. If needed can issues be raised during supervision or forwarded to the research team by the group leaders, discussed, and handled in an appropriate manner.

### Frequency and plans for auditing trial conduct {23}

No external auditing of trial conduct is planned. In Sweden, it is the Ethics Review Appeals Board that is in charge of the procedure of reviewing the conduct of trials that have been granted ethical approval by the Swedish Ethical Review Authority. The reviews take place in a random manner according to the yearly review plan of the Ethics Review Appeals Board focusing on specifically selected research areas each year.

### Plans for communicating important protocol amendments to relevant parties (e.g., trial participants, ethical committees) {25}

In the case of an important protocol amendment, all participating group leaders will be informed via email. The ethical committee will be contacted and notified about any potential protocol amendment requiring their acceptance. A major protocol amendment will also lead to an update of the protocol in the ClinicalTrials.gov registry.

### Dissemination plans {31a}

Results from the study will be presented in peer-reviewed scientific journals as well as in national reports directed to the CHCs and involved municipalities in Sweden. The results will also be presented at meetings and national and international conferences.

## Discussion

This protocol concerns a randomized controlled trial designed to evaluate the effect of a parental support program during infancy.

The process of developing the intervention and designing the study has raised several issues related to the methodology and implementation. Input from group leaders during the pilot study resulted in digital lectures to the control group instead of care-as-usual. The group leaders perceived it as unethical to recruit parents who would not be offered anything apart from usual care if allocated to the control condition. Digital lectures were also expected to increase the likelihood of retaining parents in the control group. The research group strived to make the digital lectures interesting without bringing up parental strategies. Despite this, providing the lectures could potentially reduce the differences in outcomes between the groups even though prior research shows that lectures may have no effect on parental self-efficacy [28].

The digital platform that was built for study management and data collection has high security where the CHC-nurses need an e-identification card to enter the system and the parents a social security number and the Swedish e-service BankID. However, the security of the system also entailed some challenges. Challenges connected to the use of social security numbers of the parents were met in the start of the study. Firstly, it excluded parents with protected identity. It was also an obstacle for parents that did not have a BankID and for some, there was a problem in the connection between the CSAM system and the BankID. This required rethinking from the research group to come up with technical solutions to include these parents and was considered crucial to avoid excluding parents from potentially vulnerable groups.

In the planning of the study, efforts were made to include CHCs from areas with different socio-economic backgrounds. The importance of including fathers, and not only mothers, was emphasized during the training of group leaders.

The collaboration between CHC and the municipality or other facilities from the civic society is both a strength and a challenge in the study. One advantage to the collaboration is the cross-disciplinary professions that contribute with different knowledge and experiences to the parents and to each other. A challenge is the different missions of the participating organizations that implies different preconditions for participating.

There is a lack of validated instruments to measure parenting as well as social and emotional development in infants and small children in a Swedish context. To be able to answer our research questions, there was a need to translate and validate several instruments that previously had been validated in their English versions in other settings.

The study was designed and piloted during the COVID-19-pandemic which implied some challenges. One example is that the first feasibility testing of the material with parental groups was conducted outdoors. Another challenge was that the CHC, as the whole health care system, was severely affected by the pandemic. Despite these unpredictable preconditions, all involved organizations have made great efforts to precede and participate in this research project.

This study contributes to expanding the evidence of early interventions to promote mental health and prevent ill health. Keeping in mind that mental ill-health has been identified as the biggest public health challenge in Sweden [29] and that the first years of life are crucial for future well-being [30, 31], evidence-based interventions for families with infants could be of great importance for public health.

## Trial status

Protocol version 2 of 21 February 2023. Recruitment started on 1 August 2022 and is estimated to be completed in November 2023.

## Data Availability

Access to the data will strictly be restricted to the involved researchers in line with what is stated in the ethical application. Researchers (Ph.D) with ethical permission according to Swedish law for secondary data analysis of this dataset may apply to get access of de-identified data. Written requests should be directed to the corresponding author when all data has been collected.

## Abbreviations

ACF: All Children in Focus
ASQ:SE-2: Ages & Stages Questionnaires – Socio-Emotional Second Edition
CHC: Child Health Center
CSAM: The e-health platform used in the study for randomization and data collection
PERS: Parent Emotion Regulation Scale
SDQ: Strengths and difficulties questionnaire
GDPR: General Data Protection Regulation
